# Body Condition Scores and Evaluation of Feeding Habits of Dogs and Cats at a Low Cost Veterinary Clinic and a General Practice

**DOI:** 10.1155/2016/1901679

**Published:** 2016-09-18

**Authors:** Stephanie A. Sapowicz, Deborah E. Linder, Lisa M. Freeman

**Affiliations:** Department of Clinical Sciences, Cummings School of Veterinary Medicine, Tufts University, North Grafton, MA 01536, USA

## Abstract

This study assessed body condition scores (BCS) and feeding habits for dogs and cats. Eighty-six cats and 229 dogs (and their owners) were enrolled from 2 clinics: a low cost clinic (*n* = 149) and a general practice (*n* = 166). BCS and body weight were recorded. Owners completed a survey which included animal age, sex, and breed; owner demographics; and feeding practices (e.g., diet, rationale for feeding practices). Owners from the low cost clinic had a significantly lower income (*P* < 0.001) and education (*P* < 0.001) compared to those from the general practice. Animals from the low cost clinic were younger (*P* < 0.001) and dogs were less likely to be neutered (*P* < 0.001). Overweight prevalence was 55% overall (*P* = 0.083), with a significantly higher prevalence in the general practice for cats (44% versus 66%; *P* = 0.046), but not for dogs (58% versus 53%; *P* = 0.230). Multivariate analysis showed that only neuter status was significantly associated with BCS (*P* = 0.004). Veterinarians were the most common source of nutritional information, though lack of accurate nutrition knowledge was common among all participants. These findings support the need for enhanced communication about optimal BCS and nutrition regardless of socioeconomic status.

## 1. Introduction

Pet obesity is a serious and growing concern, with up to 60% of the cat and dog population being overweight or obese [1, 2]. Guidelines for the prevention and treatment of obesity have recently been published [3]. However, effective obesity prevention and treatment require a better understanding of owner attitudes on nutrition and feeding habits so that programs can be tailored to the individual owner and animal.

A study investigating environmental factors associated with obesity in dogs showed that lower owner income and older age, as well as frequency of treats, were risk factors for overweight and obesity [2]. However, the feeding habits and reasons behind the higher risk of this population remain largely unknown. In order to effectively communicate and implement healthy weight management in cats and dogs, more information is needed to understand the feeding habits of pet owners from various income and education levels.

Therefore, the objective of the current study was to evaluate pet obesity prevalence and owner feeding habits from 2 veterinary practices: a low cost clinic and a private general practice. Results may be useful to identify potential barriers to optimal nutrition among different populations of pets and could help guide client communication and education outreach, particularly in underserved communities.

## 2. Materials and Methods

### 2.1. Study Participants

The study population included animals and their owners from 2 veterinary clinics: a private general practice veterinary clinic in central Massachusetts and a low cost veterinary clinic run by Cummings School of Veterinary Medicine at Tufts University that provides subsidized care for pets within central Massachusetts underserved communities. The low cost clinic has an income prequalification for clients, including documentation of government assistance for food or housing. All owners with animals that presented to the clinics during the study period (June–August 2013) were asked to participate. Pregnant and lactating animals and those under 1 year of age were excluded. Animals also were excluded if their owner was under 18 years of age or was not the primary caretaker for the animal. For owners who consented to participate in the study, BCS (on a 1–9 scale) was assigned by a single investigator (SAS) and body weight was recorded [4, 5]. The owners completed a survey about their pet, feeding habits, and rationale for feeding practices. The survey included questions on feeding habits, attitudes towards feeding, important sources of pet nutrition information, and owner and animal demographic information. Owner demographic information collected included the owner's age, sex, income level, and highest level of education completed, while animal demographic information included age, sex, neuter status, and breed. Questions regarding feeding practices included the type of diet fed (home-cooked, commercial dry, or commercial canned), percentage of diet coming from treats or table foods, frequency of meals, and use of supplements. Questions also were included to assess where owners obtained information about pet nutrition, how they determined the amount or type of food to feed to their pet, how many calories their pets required, and factors important in their decision to purchase a commercial pet food (full survey available upon request).

This study was approved by the Tufts University Institutional Review Board and the Cummings School Clinical Studies Review Committee.

### 2.2. Statistical Analysis

Data were assessed for normality using the Kolmogorov-Smirnov test. Since data were all normally distributed, data will be presented as mean ± SD. Categorical data were compared between the two locations using chi-square analysis, while continuous data were compared using independent *t*-tests. Multivariate analysis was performed by creating a general linear model with BCS as the outcome variable. Commercial statistical software (SPSS 22.0, IBM Corporation, Armonk, NY) was used for all analyses, and a *P* value < 0.05 was considered significant.

## 3. Results

### 3.1. Owner Demographics

Three hundred and fifteen animals and their owners were enrolled from June to August 2013: *n* = 166 from the low cost clinic and *n* = 149 from the general practice. The low cost clinic owners were younger (*P* = 0.010; Table 1), had significantly lower household income (*P* < 0.001), and completed less education (*P* < 0.001) than owners from the general practice. There was no significant difference in sex of the owners, with a predominance of females at both locations (77% overall).

### 3.2. Animal Demographics

For both dogs and cats, animals at the low cost clinic were significantly younger (dogs: *P* = 0.004; cats: *P* = 0.022; Tables 2 and 3). Dogs (*P* < 0.001), but not cats (*P* = 0.147), at the low cost clinic were more likely to be intact, with only 6 of 102 (6%) of dogs at the general practice being intact, while 46 of 117 dogs (39%) at the low cost clinic were intact. The distribution of dog breeds was different between the 2 clinics (*P* = 0.019), with the low cost clinic having more American Pit Bull Terriers and Chihuahuas. Dogs (*P* = 0.035), but not cats (*P* = 0.267), at the low cost clinic had a significantly lower body weight than those from the general practice. However, cats (*P* = 0.023), but not dogs (*P* = 0.248), at the low cost clinic had a significantly lower mean BCS. In addition, the cats at the low cost clinic had a significantly lower prevalence of overweight or obesity (i.e., BCS > 5/9; 44% at the low cost clinic compared to 66% at the general practice; *P* = 0.046). The prevalence of overweight and obesity for dogs was not significantly different between clinics (58% versus 53%; *P* = 0.230). Multivariate analysis, which included site, owner demographics, and pet demographics, showed that only neuter status was significantly associated with BCS (*P* = 0.004).

### 3.3. Feeding Habits

There were some differences in feeding habits between dog and cat owners and between the 2 clinics (Table 4). Most owners at both clinics fed primarily dry food, but the percentage of dry food was lower and the percentage of table food and home-cooked food was higher at the low cost clinic. In addition, more owners at the low cost clinic (40% total for dogs and cats) reported leaving food available at all times for their animals compared to those at the general practice (20% total for dogs and cats). However, most owners at both clinics (52%) fed their animals twice daily. Sixty-two percent of owners at both clinics (194/311) reported feeding treats at least once daily. The most common types of treats were commercial treats (*n* = 209), chews (*n* = 127), fruits/vegetables (*n* = 86), meat/cheese (*n* = 74), peanut butter (*n* = 56), and others (*n* = 30). Commercial treats were more common at the general practice, while peanut butter was more commonly provided as a treat at the low cost clinic. Most types of treats were more commonly provided to dogs compared to cats. Supplements were administered to 31/149 (21%) animals from the general practice and 21/166 (13%) animals from the low cost clinic (*P* = 0.052), with the most common supplements being joint supplements, fatty acids, and multivitamins. Joint supplements were more commonly used at the general practice compared to the low cost clinic and in dogs compared to cats. There also were species differences for types of food fed, feeding frequency, supplement use, and frequency of treats (Table 4).

Of the entire population at both sites, only 4 owners reported knowing how many calories their animal required (*n* = 2 at each site). However, 2 of the 4 responses were not sustainable for life for their animals' sizes. At both clinics, most owners reported looking for their veterinarian for advice on how much to feed [*n* = 131 (42%)], followed by the pet food feeding directions [*n* = 93 (30%)]. Twenty-one percent of owners (65/315) reported feeding an amount based on whether or not the pet looks hungry. This response was significantly more common at the low cost clinic compared to the general practice (*P* = 0.007) and for cats compared to dogs (*P* = 0.003). Veterinarians were the most frequent response as the source for nutrition information at both clinics and for both species.

The 5 most commonly reported responses for important factors in an owner's decision to select a diet for his or her pet were that the food was healthy for the pet [154/315 (49%)], ingredients [146/315 (46%)], pet preference [143/315 (45%)], cost [103/315 (33%)], and pet health needs [96/315 (31%)] (Table 5). A significantly higher proportion of owners at the low cost clinic responded that pet preference was an important factor in selecting the diet compared to the general practice (*P* < 0.001). The manufacturer's reputation was reported to be an important factor by significantly more owners at the general practice (31–39%) compared to the low cost clinic (22–27%; *P* = 0.009). There were no significant differences between clinics in the percentage of owners that chose other factors, such as cost (28–39%), availability (16–21%), convenience (6–11%), or being natural (10-11%) as a factor in choosing which diet to feed. For cat owners, pet health needs (*P* = 0.008), availability (*P* = 0.005), and convenience (*P* = 0.037) were reported more frequently as being important factors in selecting a diet compared to dog owners.

## 4. Discussion

The prevalence of overweight and obesity in the current study was high, in the range 53–58% for dogs and 44–66% for cats, depending on the site. Lack of nutritional knowledge, such as not knowing calories fed or selecting food based solely on ingredients, was common at both clinics. Multivariate analysis showed that the only variable independently associated with BCS was neuter status, with intact animals less likely to be overweight when compared to their neutered counterparts. This is not surprising since neutering is associated with an increase in appetite and a decrease in calorie requirements [6, 7]. Therefore, if veterinarians do not give specific instructions to reduce animals' calorie intake at the time of neutering or if owners are noncompliant, animals will be at higher risk for the development of overweight and obesity. Since the clinic (low cost versus general) was not significantly associated with BCS on multivariate analysis, the high rate of overweight and obesity appears to be a widespread problem that is not limited to one type of practice. Cats at the low cost clinic did have a lower prevalence of obesity compared to those at the general practice (44% versus 66%). The reason for this difference is unclear but may be related to underlying medical conditions, different relationships between people and their cats compared to dogs, or younger age of animals at the low cost clinic.

Given the high prevalence of overweight and obesity, even in the low cost clinic population in which fewer animals were neutered, higher rates might occur if more owners elected to neuter their animals. This is an important issue to consider since there is an emphasis in the United States on neutering dogs and cats. The veterinary healthcare team is a critical intervention point, and education on the importance of reducing calorie intake at the time of neutering should be emphasized. The high prevalence of overweight dogs and cats at both practices also underscores the need for veterinarians to perform nutritional screening on all animals at every visit to assess body weight, BCS, muscle condition score, and diet history [8, 9]. If animals do not have an ideal BCS (i.e., 4-5/9), the diet history usually provides important clues for sources of excess calories, and these issues should be discussed with the owner to determine the best approach to achieve safe and effective weight loss.

Results from the current study identified issues that could be specifically addressed with owners of overweight and obese animals. For example, owners at the low cost clinic were more likely to feed* ad libitum* compared to those from the general practice, a factor that may contribute to intake of excessive calories. In addition, 73% of dog owners and 30% of cat owners at both clinics responded that they gave treats, with most offering them at least once daily. Owners at the low cost clinic were also more likely to feed home-cooked foods as part of the diet. Given that the vast majority of home-cooked diets are nutritionally unbalanced [10–12], this could contribute to a nutritionally unbalanced diet in addition to excessive calories. These are issues that would be readily identified from a diet history and addressed with specific recommendations [9].

Supplements were fed to 17% of animals overall, with use being more common in dogs compared to cats. The most commonly used supplement was joint supplements, with 8% of dogs and 5% of cats using this supplement. This overall prevalence of supplement use is higher than that reported in a large multicenter study of dogs and cats conducted in the United States and Australia in which 9.9% of animals were receiving a supplement [13]. Those data were collected in 2004 so it is unclear whether the populations are different or whether there has been an overall increase in supplement use in pets. In the current study, joint supplements were more commonly used in dogs compared to cats and in the general practice compared to the low cost clinic. This may have been the result of population differences between the 2 clinics (i.e., older, large breed dogs).

Owners from both practices were similar in terms of their reasons for choosing a pet food, with both groups relying most commonly on the ingredient list, which has been shown in previous studies [13–15]. Despite a significantly lower income level for owners from the low cost clinic, there was no significant difference between clinics in the percentages of owners who reported cost or convenience as an important factor in choosing pet food. Another study found that cost was a moderately important factor in selecting pet food and that owners of overweight dogs found cost and special offers of dog food more important than owners of healthy weight dogs [15]. Pet preference in selecting a diet was also found to be an important factor in this previous study, as well as the current study, though no difference was found between owners of healthy weight and overweight dogs. The high importance placed on factors that are not evidence-based suggests that education of various populations of owners should focus on similar factors, that is, collecting diet history information at every visit, teaching owners more objective ways to select pet food than using the ingredient list or pet preference [9], and making specific recommendations for which foods and amounts to feed.

Feeding directions appear to be a particularly important area in need of owner education because many owners used the feeding directions from the pet food label (which are not always a good estimation of an individual animal's needs) or based the amount to feed on whether their animal looked hungry. Veterinarians should provide feeding instructions that help the animal to maintain an ideal BCS; this information is likely to be well accepted because owners from both clinics reported the veterinarian to be 1 of the top 3 most commonly used resources for nutrition (though less commonly at the low cost clinic). Previous studies showed similar results that pet owners perceive veterinarian advice as an important factor in feeding their pet [15]. The result from the current study that only 4 of 315 owners (1.3%) knew how many calories their animals needed [with only 2 of 315 (0.6%) being physiologically possible] suggests that owners need much more education on their pets' calorie needs, how much to feed, and other sources of calories in the diet.

Although there was a relatively large and diverse population included in the study, there are a number of differences in the animals and in the owners between clinics that could have contributed to bias. Mean body weight was higher and breeds were significantly different, which may be due to the urban versus suburban location of the low cost and general practice, respectively. In addition, owners at the low cost clinic practice had lower household incomes, received fewer years of education, and were younger than owners from the general practice. It would be useful to compare obesity rates in clinics with similar owner and animal demographics in future studies; however, in the current study, owner income, education, and age and dog age, breed, and weight were not significantly associated with BCS on multivariate analysis.

There are a number of additional limitations to the study. Animals enrolled in the study were those being presented to the 2 clinics during the study period and could include both healthy animals and those with medical conditions. Underlying medical issues could influence BCS, body weight, and diet selection so the results from the current study may not be generalizable to a population of healthy animals. However, it likely represents a typical population of animals seen by a veterinarian in general practice. Owners were asked to provide answers regarding pet food while away from their homes, so responses regarding types and amounts of food provided to their animals may not have been accurate.

Despite these limitations, the results suggest that overweight and obesity are common in at least 2 populations of dogs and cats and that this was not significantly related to age, income, and education level of the owner; to the clinic; or to animal factors, such as age or sex, other than neuter status. Most owners at both clinics used the relatively useless ingredient list to decide what to feed, instead of the guidelines set forth in recommendations by the World Small Animal Veterinary Association Global Nutrition Committee, which includes ensuring that the pet food manufacturer has a full-time qualified veterinary nutritionist and assessing the nutritional adequacy statement on the pet food to ensure the food is complete and balanced [9].

In addition, pet owners did not know how many calories their animals needed and relied on feeding directions or their perception of their animals' hunger. This suggests that there are important gaps and missed opportunities for the veterinarian and the veterinary healthcare team to be the primary resource for sound nutritional advice.

## 5. Conclusion

Overweight and obesity are common in at least 2 socioeconomic populations of dogs and cats, which was not significantly related to owner, clinic, or animal factors, other than neutering. Veterinarians were viewed as important resources for owners from both clinics, which provides an opportunity to assess pets' nutritional status and to provide accurate nutritional information to owners. This is especially important at the time of neutering since overweight and obesity were more common in neutered animals. Additional research on effectiveness of education programs is needed to prevent and treat obesity and to optimize pet health.

## Figures and Tables

**Table 1:** Summary of owner demographics from 315 animals enrolled from two veterinary clinics.

Variable	General practice	Low cost clinic	*P* value
*n*	149	166	—
Age			0.010
18–30 years	22	29	—
30–45 years	36	57	—
45–60 years	56	61	—
>60 years	32	15	—
Sex			0.157
Male	39	32	—
Female	108	130	—
Annual income			<0.001
<$10,000	2	52	—
$10,000–29,000	4	81	—
$30,000–49,000	11	17	—
$50,000–74,000	17	7	—
$75,000–100,000	20	1	—
>$100,000	80	2	—
Education			<0.001
Some high school	0	13	—
High school graduate	20	105	—
College graduate	76	40	—
Graduate degree	50	5	—

**Table 2:** Summary of dog demographic information from 229 dogs enrolled from two veterinary clinics (absolute number or mean ± SD). Only breeds reported more than 5 times were included in the table.

Variable	General practice	Low cost clinic	*P* value
*n*	107	122	—
Age (yrs)	6.8 ± 3.8	5.4 ± 3.5	0.004
Sex			<0.001
Male	52 (47 castrated)	58 (34 castrated)	—
Female	50 (49 spayed)	59 (37 spayed)	—
Breed			0.019
Mixed breed	36	43	—
Am. Pit Bull Terrier	2	13	—
Labrador Retriever	8	5	—
Chihuahua	0	9	—
Shih-tzu	3	5	—
Beagle	5	2	—
Golden Retriever	7	0	—
Pug	2	4	—
German Shepherd	3	2	—
Body weight (kg)	22.0 ± 14.9	17.9 ± 14.5	0.035
Body condition score	6.0 ± 1.2	5.8 ± 1.3	0.248
Percent overweight	62/107 (58%)	65/122 (53%)	0.230

The scale is a 1–9 scale; Am.: American.

**Table 3:** Summary of cat demographic information from 86 cats enrolled from two veterinary clinics (absolute number or mean ± SD). Only breeds reported more than 5 times were included in the table.

Variable	General practice	Low cost clinic	*P* value
*n*	42	44	—
Age (yrs)	8.3 ± 3.9	6.2 ± 4.6	0.022
Sex			0.147
Male	16 (15 castrated)	19 (10 castrated)	—
Female	25 (24 spayed)	23 (18 spayed)	—
Breed			
DSH/DLH	34	37	0.337
Body weight (kg)	5.5 ± 1.6	4.9 ± 2.7	0.267
Body condition score	6.5 ± 1.7	5.6 ± 1.8	0.023
Percent overweight	27/41 (66%)	19/43 (44%)	0.046

The scale is a 1–9 scale; DSH/DLH: domestic shorthair/domestic longhair.

**Table 4:** Summary of responses from 229 dog and 86 cat owners from two veterinary clinics (general practice, *n* = 149; low cost clinic, *n* = 166). Number of owners providing each response, with percentage in parentheses or median (range).

Variable	General practice		Low cost clinic		*P* value (clinic)	*P* value (species)
	Dogs	Cats	Dogs	Cats		
*n*	107	42	122	44	—	—
Percent food type						
Dry	96 (0–100)	97 (0–100)	90 (0–100)	80 (0–100)	0.017	0.393
Canned	0 (0–100)	1 (0–100)	0 (0–100)	15 (0–100)	0.972	<0.001
Table food	0 (0–20)	0 (0–2)	0 (0–80)	0 (0–5)	<0.001	<0.001
Home-cooked	0 (0–100)	0 (0-0)	0 (0–100)	0 (0-0)	0.011	<0.001
Commercial raw	0 (0-1)	0 (0-0)	0 (0–10)	0 (0-0)	0.075	0.540
Home-prepared raw	0 (0-0)	0 (0-0)	0 (0–10)	0 (0-0)	0.343	0.102
Feeding frequency					0.001	<0.001
*Ad libitum*	13 (12%)	16 (38%)	36 (30%)	30 (68%)	—	—
One time daily	13 (12%)	6 (14%)	21 (17%)	2 (5%)	—	—
Two times daily	77 (72%)	17 (41%)	61 (50%)	8 (18%)	—	—
Three times daily	3 (3%)	3 (7%)	4 (3%)	3 (7%)	—	—
Treats at least once daily	76 (72%)	10 (24%)	92 (75%)	16 (37%)	0.234	<0.001
Treat types^*∗*^						
Commercial	81	30	67	31	0.004	0.292
Chews	55	1	71	0	0.349	<0.001
Fruits/vegetables	37	1	47	1	0.497	<0.001
Meat/cheese	29	1	41	3	0.183	<0.001
Peanut butter	19	0	35	2	0.027	<0.001
Other	13	3	12	2	0.487	0.169
Any supplements^*∗*^	29 (27%)	2 (5%)	19 (16%)	2 (5%)	0.052	0.001
Joint supplements	17 (16%)	2 (5%)	4 (3%)	0 (0%)	<0.001	0.037
Fatty acids	9 (8%)	0 (0%)	7 (6%)	0 (0%)	0.462	0.012
Multivitamins	3 (3%)	0 (0%)	4 (3%)	0 (0%)	0.812	0.101
Probiotics	4 (4%)	0 (0%)	1 (1%)	0 (0%)	0.140	0.167
Herbal supplements	2 (2%)	0 (0%)	2 (2%)	2 (5%)	0.913	0.217
Other	0 (0%)	0 (0%)	2 (2%)	1 (2%)	0.100	0.814

^*∗*^Owners could select more than 1 answer.

**Table 5:** Summary of responses from 229 dog and 86 cat owners from two veterinary clinics (general practice, *n* = 149; low cost clinic, *n* = 166). Number of owners providing each response, with percentage in parentheses.

Variable	General practice		Low income clinic		*P* value (clinic)	*P* value (species)
	Dogs	Cats	Dogs	Cats		
*n*	107	42	122	44	—	—
Know how many calories their pet eats daily	1 (1%)	1 (2%)	1 (1%)	1 (2%)	0.927	0.450
Source for how much to feed^*∗*†^						
Veterinarian	66 (61%)	17 (41%)	38 (31%)	10 (23%)	<0.001	0.291
Product feeding directions	38 (36%)	14 (33%)	31 (25%)	10 (23%)	0.048	0.724
Pet looks hungry	12 (11%)	9 (21%)	25 (21%)	19 (43%)	0.007	0.003
Veterinarian as source for nutrition information	95 (89%)	34 (81%)	93 (76%)	32 (73%)	0.284	0.644
Factors in selecting a diet^*∗*^						
Food is healthy for the pet^‡^	56 (52%)	21 (50%)	59 (41%)	18 (41%)	0.494	0.395
Ingredients	58 (54%)	16 (38%)	55 (45%)	17 (39%)	0.264	0.460
Pet preference	33 (31%)	17 (41%)	63 (52%)	30 (68%)	<0.001	0.058
Cost	30 (28%)	13 (31%)	43 (35%)	17 (39%)	0.169	0.688
Pet health needs	35 (33%)	16 (38%)	25 (21%)	18 (41%)	0.063	0.008
Manufacturer	42 (39%)	13 (31%)	27 (22%)	12 (27%)	0.009	0.490
Availability	18 (17%)	13 (31%)	14 (12%)	13 (30%)	0.299	0.005
Natural	15 (14%)	2 (5%)	13 (11%)	4 (9%)	0.739	0.769
Convenience	6 (6%)	3 (7%)	9 (7%)	9 (21%)	0.128	0.037

^*∗*^Owners could select more than 1 answer.

^†^Only the 3 most common answers are shown.

^‡^“Healthy” was not defined for owners and could have been selected for any reason the owner thought food was healthy for the pet.

## Abbreviations

BCS: Body condition score.
